# Using Machine Learning to Evaluate the Role of Microinflammation in Cardiovascular Events in Patients With Chronic Kidney Disease

**DOI:** 10.3389/fimmu.2021.796383

**Published:** 2022-01-10

**Authors:** Xiao Qi Liu, Ting Ting Jiang, Meng Ying Wang, Wen Tao Liu, Yang Huang, Yu Lin Huang, Feng Yong Jin, Qing Zhao, Gui Hua Wang, Xiong Zhong Ruan, Bi Cheng Liu, Kun Ling Ma

**Affiliations:** ^1^ Institute of Nephrology, Zhongda Hospital, School of Medicine, Southeast University, Nanjing, China; ^2^ John Moorhead Research Laboratory, Department of Renal Medicine, University College London (UCL) Medical School, London, United Kingdom

**Keywords:** chronic kidney disease, cardiovascular disease, microinflammation, machine learning, lipid disorder

## Abstract

**Background:**

Lipid metabolism disorder, as one major complication in patients with chronic kidney disease (CKD), is tied to an increased risk for cardiovascular disease (CVD). Traditional lipid-lowering statins have been found to have limited benefit for the final CVD outcome of CKD patients. Therefore, the purpose of this study was to investigate the effect of microinflammation on CVD in statin-treated CKD patients.

**Methods:**

We retrospectively analysed statin-treated CKD patients from January 2013 to September 2020. Machine learning algorithms were employed to develop models of low-density lipoprotein (LDL) levels and CVD indices. A fivefold cross-validation method was employed against the problem of overfitting. The accuracy and area under the receiver operating characteristic (ROC) curve (AUC) were acquired for evaluation. The Gini impurity index of the predictors for the random forest (RF) model was ranked to perform an analysis of importance.

**Results:**

The RF algorithm performed best for both the LDL and CVD models, with accuracies of 82.27% and 74.15%, respectively, and is therefore the most suitable method for clinical data processing. The Gini impurity ranking of the LDL model revealed that hypersensitive C-reactive protein (hs-CRP) was highly relevant, whereas statin use and sex had the least important effects on the outcomes of both the LDL and CVD models. hs-CRP was the strongest predictor of CVD events.

**Conclusion:**

Microinflammation is closely associated with potential CVD events in CKD patients, suggesting that therapeutic strategies against microinflammation should be implemented to prevent CVD events in CKD patients treated by statin.

## Introduction

In the death of patients with chronic kidney disease (CKD), cardiovascular events are the uppermost factors. Dyslipidaemia has been proved to be one leading risk factor for cardiovascular disease (CVD) in CKD patients (1, 2). Therefore, hyperlipidaemia has been a main target for statin therapy in CKD patients. However, some studies have revealed a U-shaped relationship between the plasma cholesterol level and CVD mortality among patients with end-stage renal disease (ESRD), indicating an increase in mortality related to CVD when the plasma low-density lipoprotein (LDL) level declined to normal or below normal (3). This result suggests that ESRD patients with low plasma cholesterol levels (within or less than the normal range) tend to suffer a more severe atherosclerotic burden (4). Furthermore, AURORA and 4D clinical trials both demonstrated that statins lowered the plasma LDL cholesterol level of ESRD patients undergoing maintenance dialysis but had no significant effect on CVD events (5, 6). The mechanisms leading to this phenomenon have still not been elucidated. Some studies have reported that the plasma cholesterol levels are negatively correlated with CVD events in ESRD patients, which could be ascribed to the cholesterol-lowering effect of systemic microinflammation. Interestingly, the JUPITER clinical trial demonstrated that strict control of the level of hypersensitive C-reactive protein (hs-CRP) can reduce CVD events (7). Recently, the CANTOS clinical trial further demonstrated that the chances of continual CVD events decreased drastically by the anti-inflammatory therapy targeting the interleukin-1β innate immunity pathway. What’s more, the lipid-lowering effects were excluded in this study (8). We previously demonstrated microinflammation-induced cholesterol redistribution from peripheral tissues to the liver and from extracellular to intracellular regions, resulting in hypolipidaemia and accelerating kidney injury, atherosclerosis, and nonalcoholic fatty liver disease (9–13). This redistribution was mainly caused by microinflammation-mediated disruption of cholesterol homeostasis, which is characterized by increased cholesterol uptake modulated by the LDL receptor and CXC chemokine ligand 16 (CXCL16) pathways and the reduction of cellular cholesterol efflux mediated by the ATP-binding cassette transporter A1 (*ABCA1)* pathway (9–13).

Microinflammation refers to chronic, persistent, low-grade existing inflammation that is not the result of infections by pathogenic microorganisms. The abovementioned series of studies show that microinflammation plays crucial role in the occurrence of CVD events in CKD patients. However, more clinical data is needed to confirm the possible synergistic effects of microinflammation with lipid metabolism disorder and the therapeutic effect of statins on CVD in CKD patients under the condition of microinflammation.

Hs-CRP, as a sensitive indicator of inflammation, has been proved to be not only the independent risk factor of cardiovascular disease but also mediator of atherosclerosis through IL-1-to-IL-6-to-CRP signalling pathway by CANTOS trial (8). Sub-analysis of FOURIER trials reveals a stepwise risk process in accordance with the baseline values of hs-CRP and no correlation between dramatic reductions of LDL-C with status for hs-CRP (14). Consequently, although the causal relationship between hs-CRP and LDL-C as well as CVD events is still questionable, hs-CRP represents a major biomarker of inflammation and risk factor in CVD.

Machine learning (ML) is an important branch of artificial intelligence (AI) in which computer systems are used to learn algorithms and statistical models from sample statistics and past experience. Current popular machine learning algorithms include the support vector machine (SVM) (15), random forest (RF) (16), logistic regression (LR), K nearest neighbours (KNN), and neural network (NN) (17). Machine learning has been widely used in disease prediction (18), patient categorization (19), computer-assisted diagnosis, and genomic medicine (20).

Therefore, this study was aimed to establish prediction models for LDL and CVD events using machine learning algorithms and evaluate the association among microinflammation, the plasma LDL level, and CVD events in CKD patients to provide evidence for making clinical decisions.

## Materials and Methods

### Data Collection of CKD Patients

The electronic medical record (EMR) system in Zhong Da Hospital has been in operation since 2013, and 11932 EMRs of patients hospitalized in the renal department are available up to September 2020. According to the inclusion criteria of this study, we extracted a total of 2578 EMRs of CKD patients with hs-CRP results. We found complete data for 914 cases of EMRs, including serum creatinine (Scr), body mass index (BMI), serum alanine transaminase (ALT), serum aspartate transaminase (AST), serum albumin, total cholesterol (TC), blood glucose, haemoglobin, erythrocyte sedimentation rate (ESR), systolic blood pressure, diastolic blood pressure, white blood cells, age, sex, and use of statins. The types of statins used were not subdivided in this study. All patients included in this study were not treated with ezetimibe. All subjects signed the written informed consent. The study was approved by the ethics committee of Zhongda hospital, Southeast University. For protection, all private information was removed before publication.

### The Definition of CKD and CVD Events in This Study

The data extracted from the EMR system were used to screen patients diagnosed with CKD or ESRD based on the International Classification of Diseases (ICD) code (**Supplementary Table 2**). The CVD events referred in this study mainly include the following cardiovascular end points: cardiovascular death, non-cardiovascular death, unknown cause of death, myocardial infarction, unstable angina pectoris, heart failure events, percutaneous coronary intervention, peripheral vascular intervention, stent thrombosis. In addition, cardiac dysfunction and myocardial injury are taken as potential CVD events. Indices reflecting cardiac function, including creatine kinase isozyme (CK-*MB*), *myoglobin (MYO)*, and *troponin I (TnI)*, were collected. We analysed these cardiac-related indices to elucidate the effects of microinflammation on CVD events in statin-treated CKD patients.

### Establishment of Predictive Models

The laboratory results were used to develop predictive models to analyse potential indicators of lipid profiles and CVD events. To elucidate the effect of microinflammation on these results, two sets of models for use in machine learning were established, and the Gini points were evaluated. With the support of ML, we managed predicting models with data after imputation. The mean value was taken to make up for imputation. 751 EMRs, accounting for 30% of the aggregate data, were randomly selected as the test set. 70% of the aggregate data, including the remaining complete data and the data after imputation, were used as training set.

### Label Definitions

Continuous variables and indices of drug usage were treated as classified variables for modelling purposes. The details are presented here. (1) LDL: Three levels were assigned based on the clinical normal LDL value: 0 for an LDL above 3.62, 2 for an LDL less than 2, and 1 for all other LDL values. (2) Use of statins: Doctor-recommended statin use was labelled 1, and other statin use was labelled 0. (3) Sex: Females were labelled 1, and males were labelled 0. (4) CVD-related indices: One (or more) abnormal indices out of the three CVD indices was marked as ‘positive’, and other cases were marked as ‘negative’.

### Determination of eGFR

The estimated glomerular filtration rate (eGFR) was calculated by a simplified formula for the modification of diet in renal disease *(*MDRD*).*


$$\text{eGFR}=186\times (\text{Scr})-1.154\times (\text{age})-0.203\times {\left(0.742\text{ female}\right)}^{\left[14\right]}$$

$$\text{eGFR}:(\text{ml/}\min/1.73{\text{m}}^{2});\text{Scr}:(\text{mg/dl})$$

### Supervised Learning Classifiers

RF, LR, SVM, NN, and KNN were applied using a Python code to predict the LDL level and CVD-related indices, including TnI, CK-MB, and MYO. All the factors were included in the models. The LDL level and CVD were classified to make the prediction a classification task. The models were evaluated in terms of the accuracy and area under the curve (AUC). A fivefold cross validation method was employed to prevent the problem of overfitting.

### Statistical Analysis

Demographic, clinical, and treatment characteristics were retrospectively analysed. Continuous data were represented as means, and nonnormally distributed data were represented as medians and interquartile ranges and the categorical data were reported as number and percentage. Disaggregated data were expressed as figures and percentages. Python 3.7.6 (https://docs.python.org/3.7) and StataMP software (Version Stata 16 MP) were used to perform the analysis.

## Results

### Study Population

**Figure 1:**
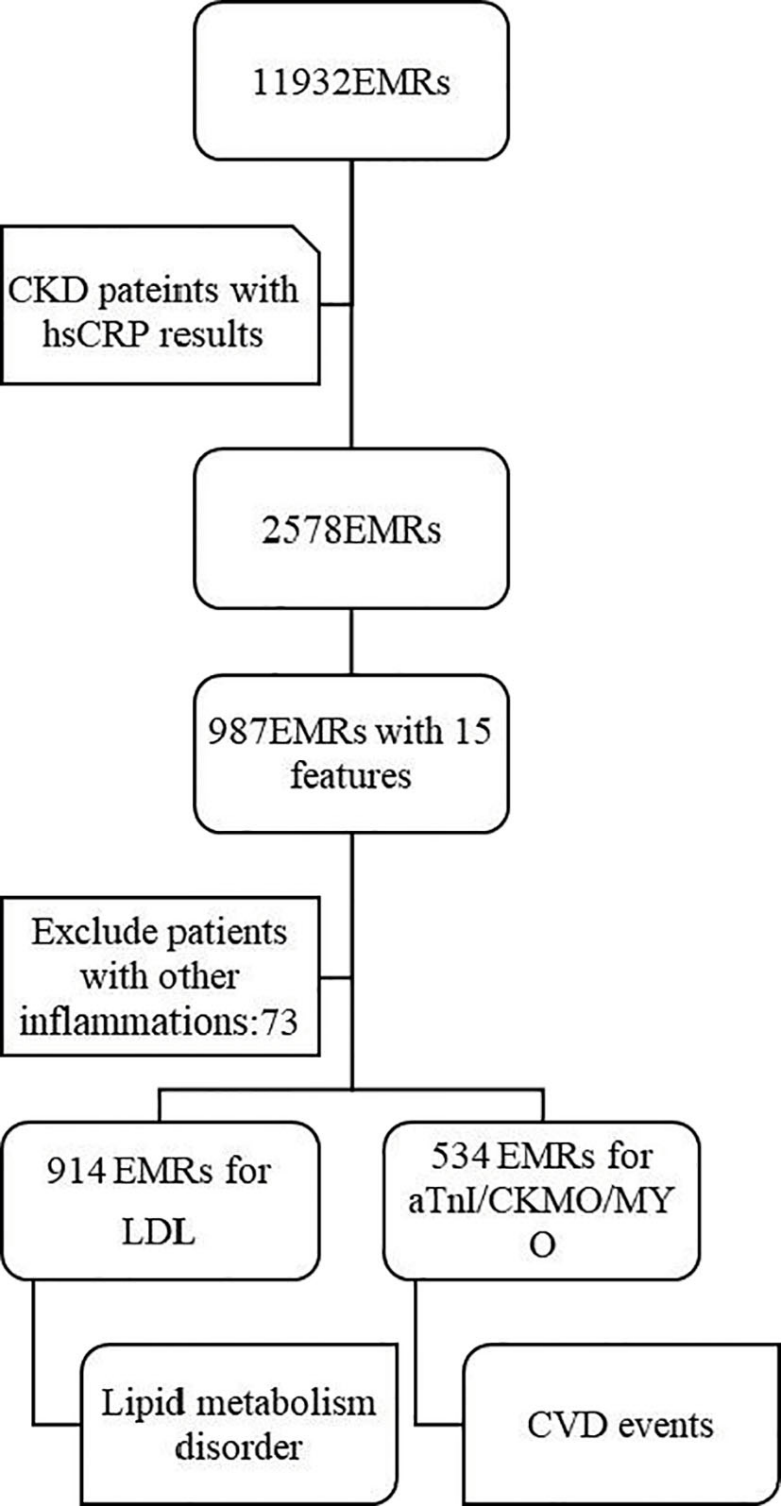
Study population. From the initial database with 11932 EMRs, 2578 EMRs of CKD patients with hsCRP results were selected firstly. Then, among the 987 EMRs with complete 15 features included, 73 EMRs were excluded. Finally, 914 EMRs and 534 EMRs were used for prediction for lipid metabolism disorder and CVD events respectively. EMRs, Electronic Medical Record; CKD, Chronic kidney disease; hsCRP, hypersensitive C-reactive protein; LDL, low-density lipoprotein; CK-MB, creatine kinase isozyme; MYO, myoglobin; aTnI, Troponin I; CVD, cardiovascular disease.

A total of 11932 EMRs were collected, of which 2578 EMRs were diagnosed as CKD with hs-CRP determination reports. Based on clinical experience, 15 features were selected as variables for the prediction model. After deleting any entries with missing data, a total of 914 EMRs were finally included. In addition, 534 of these 914 EMRs had indicators reflecting heart function, including TnI, CK-MB, and MYO (**Figure 1**).

### Clinical Characteristics of CKD Patients

The 914 EMRs were used to establish an LDL prediction model. The median age of the LDL cohort was 61, and 58.75% of the patients were male. The median eGFR of the patients enrolled in the LDL cohort was 9.01 ml/min/1.73 m^2^ (interquartile range: 5.99-16.92 ml/min/1.73 m^2^). A total of 58.53% of the patients had low-density lipoprotein ranging between 2 and 3.62 mmol/L, which was classified as level 1. Other demographic descriptions, clinical characteristics, and statin treatment methods are shown in **Table 1**.

**Table 1:** Clinical characteristics of patients in predicting model of LDL.

Demographic/clinical characteristics		N=914
Gender, n,%	Male	537 (58.75)
	Female	377 (41.25)
Age, year		61 [49-72]
Body Mass Index, kg/m^2^		23.49 [20.83-26.56]
Systolic pressure, mmHg		140 [127-158]
Diastolic pressure, mmHg		80 [70-90]
Laboratory data		
Low density Lipoprotein-C, mmol/L		2.38 [1.89-2.96]
	0 (>3.62)	91/914 (9.96%)
	1 (2-3.62)	535/914 (58.53%)
	2 (<2)	288/914 (31.51%)
High sensitive C-reactive protein, mg/L		3.38 [0.98-13.58]
Serum Creatinine, umol/L		532 [307.5-757.25]
Serum Alanine Transaminase, IU/L		13 [9-21]
Serum Aspartate Transaminase, IU/L		16 [12-20]
Plasma Albumin, g/L		37.5 [33.5-41]
Total Cholesterol, mmol/L		4.14 [3.43-4.96]
Blood Glucose, mmol/L		5.18 [4.6-6.42]
Haemoglobin, g/L		101 [86.25-116]
Erythrocyte Sedimentation Rate, mm/h		33 [19-60]
White Blood Cell, 10^9/L		6.01 [4.85-7.53]
eGFR, ml/min/1.73 m^2^		9.01 [5.99-16.92]
Treatments		
Use of stain, n, %	Yes	198/914 (21.66%)
	No	716/914 (78.34%)

Of the 914 EMRs, 380 records were deleted because of the absence of data on cardiovascular function. Finally, 534 EMRs were selected to predict CVD events. The demographic description, clinical characteristics, and statin treatment used are shown in **Table 2**. A total of 58.61% of the CVD cohort was male, which was similar to the LDL cohort. The median hs-CRP and ESR of the CVD cohort was 4.64 mg/L and 35 mm/L, respectively, which were clearly higher than those of the LDL cohort. In addition, a higher percentage of patients were treated with statins in the CVD cohort (26.03%) than in the LDL cohort (21.66%).

**Table 2:** Clinical characteristics of patients in predicting model of CVD.

Demographic/clinical characteristics		N=534
Gender, n, %	Male	313/524 (58.61)
	Female	221/534 (41.39)
Age, year		63 [51-73]
Body Mass Index, kg/m^2^		23.51 [20.90-26.72]
Systolic pressure, mmHg		141 [127-160]
Diastolic pressure, mmHg		80 [70-90]
Low density Lipoprotein-C, mmol/L		2.35 [1.85-2.95]
Highly sensitive C-reactive protein, mg/L		4.64 [0.86-17.4]
Serum Creatinine, umol/L		537.5 [349-733]
Serum Alanine Transaminase, IU/L		13 [9-20]
Serum Aspartate Transaminase, IU/L		16 [12-21]
Plasma Albumin, g/L		36.65 [33-40.1]
Total Cholesterol, mmol/L		4.08 [3.37-4.89]
Blood Glucose, mmol/L		5.35 [4.68-6.89]
Haemoglobin, g/L		97.5 [84-114]
Erythrocyte Sedimentation Rate, mm/h		35 [20-66]
White Blood Cell, 10^9/L		6.12 [4.87-7.66]
eGFR, ml/min/1.73 m^2^		9.01 [6.13-14.25]
Treatments		
Use of stain, n, %	Yes	139/534 (26.03%)
	No	395/534 (73.97%)
CVD related data	N	
CKMB, IU/L	173	11.50 [7.1-19.1]
MYO, ng/mL	44	268.5 [177-405.9]
aTnI, ng/mL	519	0.024 [0.01-0.05]

CKMB, creatine kinase-MB; MYO, Myoglobin; aTnI, Troponin I.

### LDL Prediction Model

**Figure 2:**
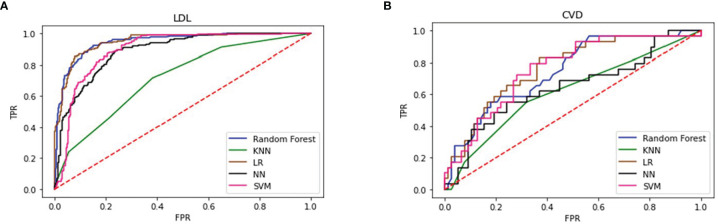
Performance of 5 types of predicting models of LDL **(A)** and CVD **(B)** LDL, low-density lipoprotein; CVD, cardiovascular disease; KNN, K Nearest Neighbors; LR, logistic regression; NN, neural network; SVM, support vector machine.

**Figure 3:**
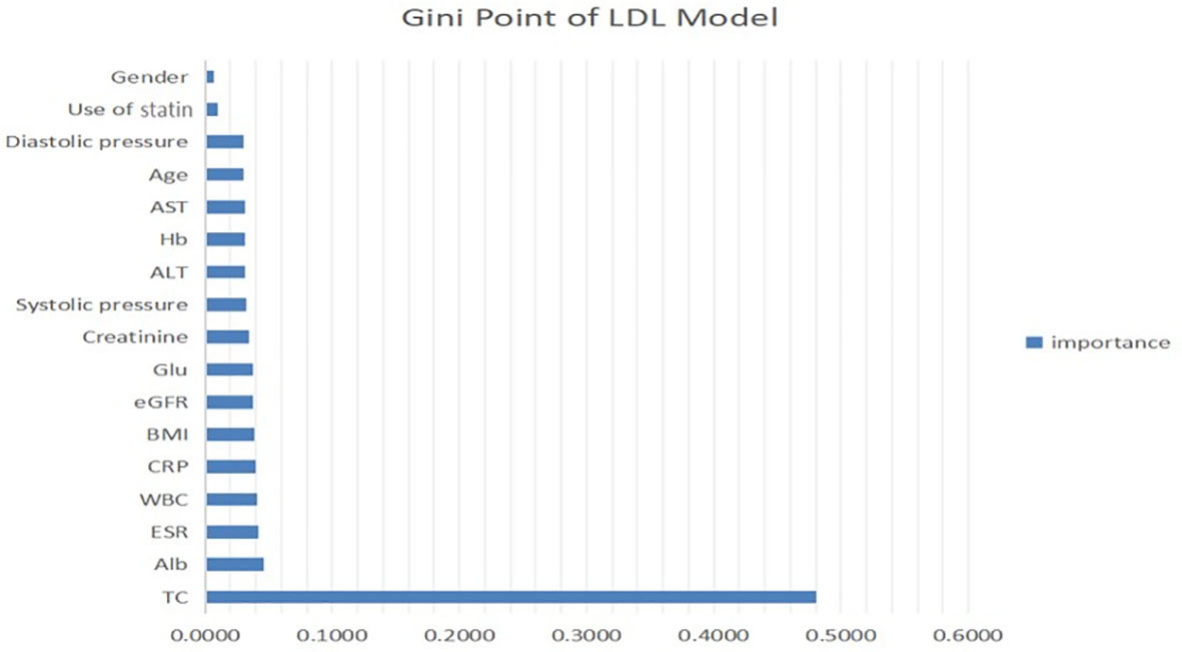
Contribution of predictors of LDL level in CKD patients with micro-inflammatory. ALT, serum alanine transaminase; AST, serum aspartate transaminase; Alb, serum albumin; TC, total cholesterol; Glu, blood glucose; Hb, haemoglobin; ESR, erythrocyte sedimentation rate; BMI, body mass index; WBC, white blood cells; hsCRP, hypersensitive C-reactive protein; eGFR, estimated glomerular filtration rate.

**Figure 2A** shows that the RF and LR were the best performing models. The models developed using RF and LR had comparable accuracies (82.27% and 82.71%, respectively) and the same AUC of 0.94. In addition, we determined an importance ranking by computing the Gini index using the RF algorithm. All factors were included in the establishment of RF model. The importance ranking results are shown in **Figure 3**. Among the various predictors, TC was the most important for the analysis, and the use of statins and sex were the least important. Except for TC, hs-CRP had a significant influence on the outcome.

### CVD Prediction Model

We trained 5 types of models similarly to predict CVD-related indices. **Table 3** shows that RF was the best performing model, with an accuracy of 74.15% and an AUC of 0.69. The LR model exhibited the second-best performance, with an accuracy of 73.4% and an AUC of 0.69. **Figure 2B** shows that four models had the same AUC of 0.69 (whereas KNN had a different AUC), which indicated that these four models had strong prediction ability.

**Table 3:** Comparison of validation result of LDL and CVD models.

Outcome	Algorithms	Accuracy (%)	AUC
LDL	Random forest	82.27%	0.94
	KNN	51.86%	0.71
	SVM	78.44%	0.90
	Logistic Regression	82.71%	0.94
	NN	63.89%	0.82
CVD	Random forest	74.15%	0.69
	KNN	69.85%	0.55
	SVM	72.46%	0.69
	Logistic Regression	73.40%	0.69
	NN	61.06%	0.59

SVM, support vector machine; KNN, k nearest neighbors; NN, Neural Networks.

**Figure 4:**
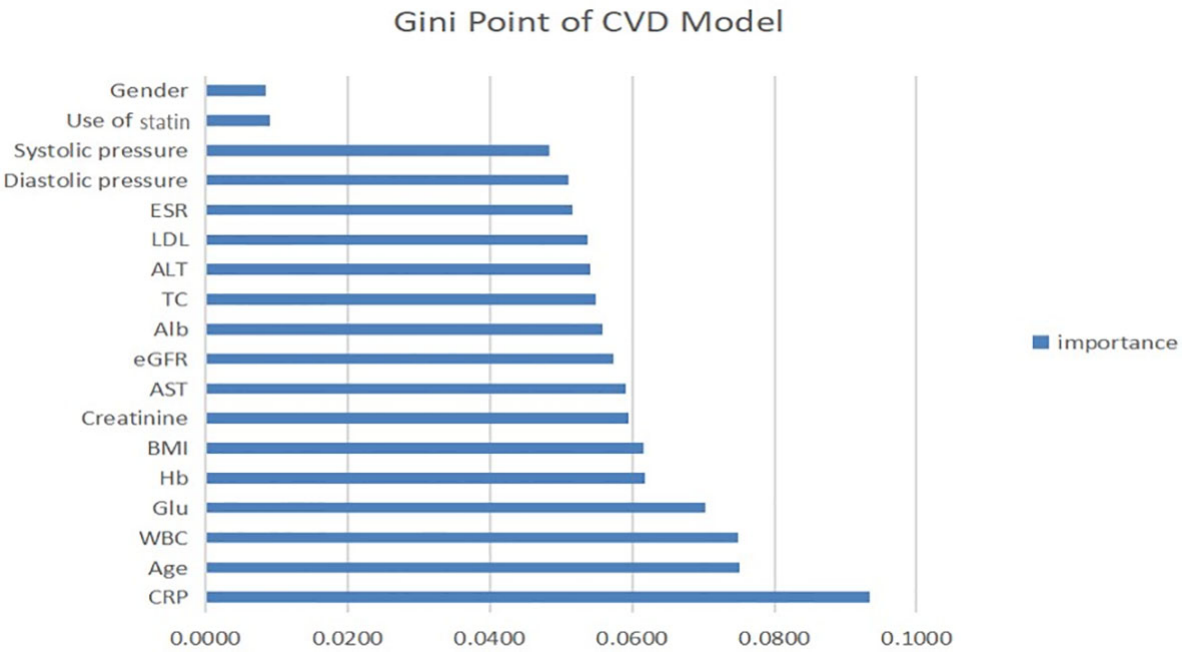
Contribution of predictors of CVD index in CKD patients with micro-inflammation. ALT, serum alanine transaminase; AST, serum aspartate transaminase; Alb, serum albumin; TC, total cholesterol; Glu, blood glucose; Hb, haemoglobin; ESR, erythrocyte sedimentation rate; BMI, body mass index; WBC, white blood cells; hsCRP, hypersensitive C-reactive protein; eGFR, estimated glomerular filtration rate.

Similarly, the Gini point of the predictors in the CVD model was evaluated and is presented in **Figure 4**. As for the LDL model, the use of statins and sex were the least important predictors. hs-CRP was the strongest predictor of CVD events using this model and therefore had the highest impact on the outcome. Age, white blood cells, and blood glucose were very fairly important predictors.

### Performance Comparison of Five Models

Five supervised classification models were generated to predict LDL and CVD events. **Table 3** shows the cross-validation results obtained using each algorithm. RF and LR exhibited the best performance for the LDL model with an AUC of 0.94 and high accuracy. For the CVD model, random forest exhibited the highest accuracy of 74.15%, and the AUCs of RF, LR, and SVM were almost the same, indicating that these models had the same prediction performance. However, KNN performed poorly for both the LDL and CVD models, with AUCs of 0.71 and 0.55, respectively.

### Performance Comparison of Models Before and After Imputation

The performance of models improved slightly according to performance of the AUC-ROC (**Supplementary Figure 1**) and accuracy, especially in algorithms like RF (from 82.27% to 83.61%) and LR (from 82.27% to 84.02%) after imputation (**Supplementary Table 1**). There were some slight changes in the importance rankings of predictive models. Nonetheless, the ranking of valued labels, including use of statin (**Supplementary Figure 3**) and hs-CRP, did not change in the LDL models. Meanwhile, ‘hs-CRP’ stayed in the first place and ‘use of statin’ (**Supplementary Figure 2**) remained falling behind, from the penultimate to tailender.

## Discussion

CKD is an independent hazard factor for coronary disease (21). LDL cholesterol is the main therapeutic target among lipoproteins leading to dyslipidaemia in CKD patients. Statins are inhibitors of 3-hydroxy-3-methylglutaryl-CoA reductase (HMGR) and mainly inhibit endogenous cholesterol synthesis. However, some studies have shown very limited benefits of using statins as lipid-lowering therapy to prevent CVD events in the CKD cohort, especially under microinflammatory conditions. Storey et al. studied the relationship between CRP and CVD events in the Sharp trial (22). The results obtained based on more than 2.5 years of follow-up showed that a 3-fold increase in CRP was associated with a 28% increase in the occurrence of major CVD events. Thus, microinflammation not only accelerated the progression of CVD in patients with CKD but also weakened the effect of statins as lipid-lowering therapy. However, these results need to be confirmed by more clinical data, especially from cohorts of Chinese CKD patients.

Through this study, a prediction model for LDL levels was established with a variety of indicators, including microinflammation and lipid-lowering treatment. The total cholesterol level had the highest importance ratio, whereas statin use was the second-least important predictor and was not even as influential as blood pressure. This result could be related to microinflammation, because the lipid-lowering effect of statins is considerably reduced under microinflammatory conditions. Furthermore, the LDL prediction model showed that the plasma total cholesterol level of CKD patients had the strongest correlation with LDL, which could be attributed to lipid metabolism disorder in CKD patients. Hs-CRP had a high importance rank, indicating that microinflammation plays a more important role in the modulation of lipid metabolism disorder in CKD patients than in LDL patients. These findings suggest that more attention should be given to anti-microinflammation therapy rather than simply reducing the plasma cholesterol levels of CKD patients.

Second, we established a CVD prediction model to explore the correlation between microinflammation and CVD in CKD patients. The accuracy and AUC of the CVD models were less than 75% and 0.7, respectively. We determined the importance rank of each feature in the CVD model. Hs-CRP was the most important predictor, and statin use and the LDL level were relatively less important, suggesting that microinflammation is more important than the blood lipid level in the progression of CVD in patients with CKD. Combining the results of previous studies with those obtained using our LDL model shows that microinflammation has a dual effect (on the blood lipid level and the onset of CVD events) and is therefore the most important risk factor.

FOURIER trails provided robust evidence for CVD prevention as LDL-C lowering of evolocumab was consistent across CKD groups without impacts on hs-CRP values (23). However, combining the positive correlation between hs-CRP and CVD events in patients who achieving relatively lower LDL-C levels (14) with our findings in this study, an inference could be drawn that inflammation could be the actual principal factor when LDL-C is brought at target. Due to the limitations of our research types, more prospective studies are needed to prove this deduction scrupulously.

In addition to LDL-C, studies on HDL also have led an important direction for the inverse association between plasma HDL-C concentration and the risk of CVD. However, due to the high complexity of HDL particles, the causal relationship between HDL-C and the increasing risk of CVD events in CKD patients remains controversial (24). Untersteller et al. showed that neither the quantity of HDL-C nor the composition or function of HDL-C could predict cardiovascular outcome among CKD patients independently (25). Shao et al. found that the protein cargo of HDL-C could be used as a biomarker and mediator for elevated CVD risk in CKD patients (26). Calabresi et al. reported that acquired plasma lecithin cholesterol acyltransferase (LCAT) deficiency was a major cause of low plasma HDL levels in patients with CKD, making it an attractive therapeutic target to reverse dyslipidaemia and reduce potential cardiovascular events (27). Although HDL-C was not included in this study, we thought that more studies are needed to promote our understanding of the progression and occurrence of CVD in patients with CKD.

To evaluate the application of machine learning to clinical databases, the accuracy and AUC of the models were compared using five common algorithms. The random forest had the best performance, followed by LR and SVM, whereas KNN and NN had the poorest and unstable performance.

The aforementioned results could be correlated with the type of data used in this study. Digital data were used in this study, whereas the NN is mainly used for image information processing. Therefore, RF and LR are preferred for clinical data processing. In this process, we thought that the amount of data with all the features was sufficient for model training, and feature filling would destroy the original distribution of data, which was shown in the change order of features after interpolation. Therefore, we used the complete data for model training, and the results were still satisfactory.

Immune cells play an important role in maintaining kidney homeostasis and dealing with renal injury. The destruction of body balance can quickly trigger the metabolic reorganization of kidney immune cells and non-immune cells, which results in microinflammation and tissue damage (28). The susceptibility of CKD patients may contribute to microinflammation, leading to dyslipidemia and increasing the incidence of CVD events. For instance, Chen et al. demonstrated that microinflammation weakened the therapeutic efficacy of statins through the destruction of 3-hydroxy-3-methylglutaryl-CoA reductase feedback regulation (29, 30). Our results in this study demonstrated the correlation between microinflammation and dyslipidemia as well as CVD events in CKD patients. However, the specific mechanisms need to be further studied in the future work.

This study had some limitations. The sample size was not sufficiently large. Some samples were excluded due to incomplete data. Although the model performed well in internal validation, external validation is needed to confirm the results. In addition, this study was cross-sectional and therefore had limited utility for exploring the association among microinflammation, lipid metabolism disorder, lipid-lowering treatment and CVD events, such that a clear causal relationship was not determined.

In summary, we successfully established prediction models for the LDL level and CVD events using five common algorithms, among which RF and LR were proven to be the most suitable for clinical data processing. An analysis of the Gini ranking showed that microinflammation was highly relevant to lipid metabolism disorders in CKD patients and CVD events, which raises concerns about anti-inflammatory treatment. The limited benefit of lipid-lowering treatment under microinflammation warrants further consideration of lipid management for CKD patients.

## Data Availability Statement

The original contributions presented in the study are included in the article/**Supplementary Material**. Further inquiries can be directed to the corresponding author.

## Ethics Statement

The studies involving human participants were reviewed and approved by the Ethics Committee of Zhongda Hospital, Southeast University. The patients/participants provided their written informed consent to participate in this study.

## Author Contributions

KM and XL designed the work. XL, TJ, MW, WL, YH, YLH, FJ, and QZ collected and integrated the data. XL and TJ analyzed the data and prepared the manuscript. XL, XR, BL, and KM edited and revised the manuscript. All authors contributed to the article and approved the submitted version.

## Funding

This study was supported by the National Natural Science Foundation of China (82170736, 81970629), the Jiangsu Province Social Development Project (BE2018744), the Project for Jiangsu Provincial Medical Talent (ZDRCA2016077), the Fundamental Research Funds for the Central Universities (3224002110D), and the Jiangsu Province Ordinary University Graduate Research Innovation Project (SJCX20-0055).

## Conflict of Interest

The authors declare that the research was conducted in the absence of any commercial or financial relationships that could be construed as a potential conflict of interest.

## Publisher’s Note

All claims expressed in this article are solely those of the authors and do not necessarily represent those of their affiliated organizations, or those of the publisher, the editors and the reviewers. Any product that may be evaluated in this article, or claim that may be made by its manufacturer, is not guaranteed or endorsed by the publisher.

## Supplementary Material

Supplementary Figure 1 Performance of 4 types of predicting models of LDL and CVD after imputation. LDL, low-density lipoprotein; CVD, cardiovascular disease; KNN, K Nearest Neighbors; LR, logistic regression; NN, neural network.

Supplementary Figure 2 Contribution of predictors of LDL level in CKD patients with micro-inflammatory after imputation. ALT, serum alanine transaminase; AST, serum aspartate transaminase; Alb, serum albumin; TC, total cholesterol; Glu, blood glucose; Hb, haemoglobin; ESR, erythrocyte sedimentation rate; BMI, body mass index; WBC, white blood cells; hsCRP, hypersensitive C-reactive protein; eGFR, estimated glomerular filtration rate.

Supplementary Figure 3 Contribution of predictors of CVD index in CKD patients with micro-inflammation after imputation. ALT, serum alanine transaminase; AST, serum aspartate transaminase; Alb, serum albumin; TC, total cholesterol; Glu, blood glucose; Hb, haemoglobin; ESR, erythrocyte sedimentation rate; BMI, body mass index; WBC, white blood cells; hsCRP, hypersensitive C-reactive protein; eGFR, estimated glomerular filtration rate.
