# Supplementary figures and images for: Using Machine Learning to Evaluate the Role of Microinflammation in Cardiovascular Events in Patients With Chronic Kidney Disease
Source: Front Immunol. 2022 Jan 10;12:796383. doi: 10.3389/fimmu.2021.796383 (PMC8784809; doi:10.3389/fimmu.2021.796383)

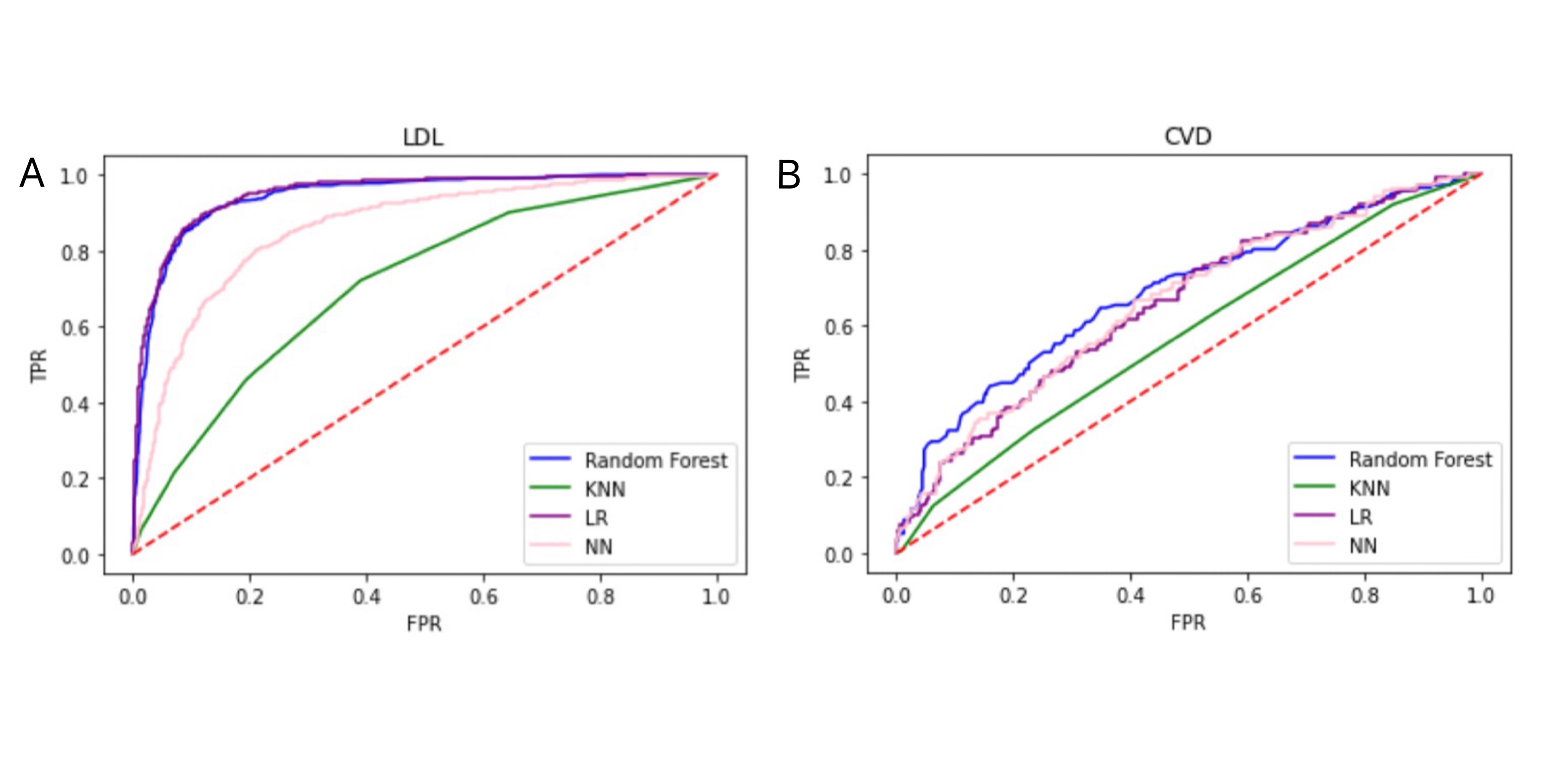

Supplement: Supplementary Figure 1 — Performance of 4 types of predicting models of LDL and CVD after imputation. LDL, low-density lipoprotein; CVD, cardiovascular disease; KNN, K Nearest Neighbors; LR, logistic regression; NN, neural network. [file Image_1.tif]

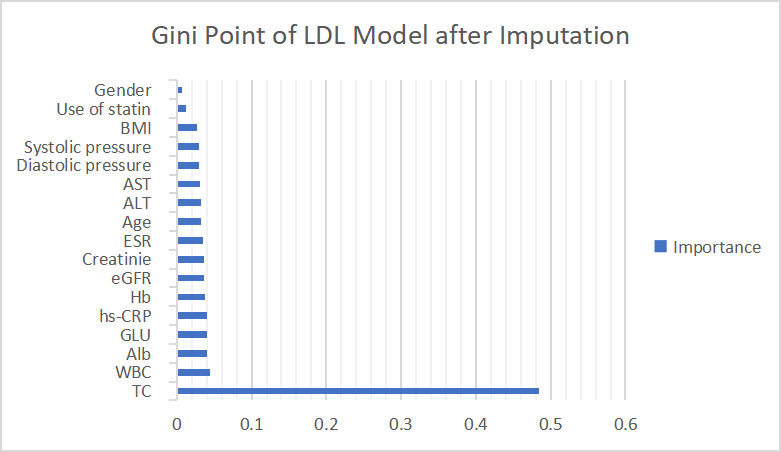

Supplement: Supplementary Figure 2 — Contribution of predictors of LDL level in CKD patients with micro-inflammatory after imputation. ALT, serum alanine transaminase; AST, serum aspartate transaminase; Alb, serum albumin; TC, total cholesterol; Glu, blood glucose; Hb, haemoglobin; ESR, erythrocyte sedimentation rate; BMI, body mass index; WBC, white blood cells; hsCRP, hypersensitive C-reactive protein; eGFR, estimated glomerular filtration rate. [file Image_2.tif]

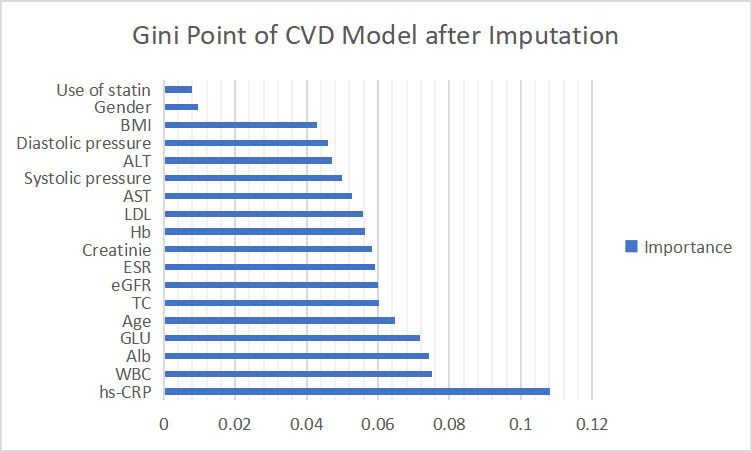

Supplement: Supplementary Figure 3 — Contribution of predictors of CVD index in CKD patients with micro-inflammation after imputation. ALT, serum alanine transaminase; AST, serum aspartate transaminase; Alb, serum albumin; TC, total cholesterol; Glu, blood glucose; Hb, haemoglobin; ESR, erythrocyte sedimentation rate; BMI, body mass index; WBC, white blood cells; hsCRP, hypersensitive C-reactive protein; eGFR, estimated glomerular filtration rate. [file Image_3.tif]
