# Supplementary material for: Using Machine Learning to Evaluate the Role of Microinflammation in Cardiovascular Events in Patients With Chronic Kidney Disease
Source: Front Immunol. 2022 Jan 10;12:796383. doi: 10.3389/fimmu.2021.796383 (PMC8784809; doi:10.3389/fimmu.2021.796383)
Supplement: Supplementary file 4 [file Table_1.pdf]

Supplementary data

Table1: The new validation result of LDL and CVD models after imputation

Outcome	Algorithms	Accuracy (%)	AUC
LDL	Random forest	83.61%	0.94
	KNN	53.72%	0.71
	Logistic Regression	84.02%	0.95
	NN	59.23%	079
CVD	Random forest	72.25%	0.69
	KNN	63.44%	0.57
	Logistic Regression	69.38%	0.66
	NN	68.50%	0.63

KNN, k nearest neighbors; NN, Neural Networks.

Table2: The ICD-10 codes of CKD and CVD referred in this study

Disease	ICD codes
CKD	N18.902,N18.905,N18.003,N18.807,N18.001,N18.801,N18.802,N18.803,N18.804,N18.8011,N18.002,N18.8012,N18.8014, N18.8013
CVD	Z35.209,I21.900x011,I20.005,I20.006,I21.001,I21.002,I21.003,I21.004,I21.100,I21.100x002,I21.100x003,I21.103,I21.104,I21.105,I21.106,I21.200,I21.200x003,I21.200x009,I21.200x010,I21.200x014,I21.200x015,I21.200x016,I21.200x017,I21.200x018,I21.200x019,I21.200x020,I21.200x021,I21.200x022,I21.200x023,I21.200x024,I21.200x025,I21.200x026,I21.200x027,I21.200x028,I21.200x029,I21.200x030,I21.200x043,I21.204,I21.205,I21.206,I21.207,I21.208,I21.210,I21.211,I21.212,I21.213,I21.300,I21.300x004,I21.400,I21.400x001,I21.400x004,I21.401,I21.900,I21.900x001,I21.900x012,I21.900x013,I21.900x014,I21.900x016,I21.900x017,I21.400x011,I21.300x011,I21.000x002,I21.000x001,I21.000x003,I21.200x042,I21.100x001,I21.200x041,I20.000,I20.900,N18.800x020,I50.000,I50.900x017,I50.900x019,I50.907,I50.908,Z95.501,Z95.800x003,T82.804,I50.900x002,I50.906
